# Construction and validation of a predictive model for hypothermia complication during endoscopic thyroidectomy for thyroid cancer

**DOI:** 10.3389/fmolb.2025.1758239

**Published:** 2026-01-12

**Authors:** Hui Ye, Lu Xia, Tian Zhan, Haiwei Zhang

**Affiliations:** 1 Department of Anesthesiology and Perioperative Medicine, The First Affiliated Hospital of Nanjing Medical University, Nanjing, Jiangsu, China; 2 Department of General Surgery, The Affiliated Cancer Hospital of Nanjing Medical University, Nanjing, Jiangsu, China

**Keywords:** endoscopic thyroidectomy, hypothermia complication, predictive model, risk factors, thyroid cancer

## Abstract

**Background:**

Intraoperative hypothermia frequently occurs during surgery and can negatively impact patient outcomes. The study focuses on establishing a clinical prediction model to identify the risk of intraoperative hypothermia in patients undergoing endoscopic thyroidectomy for thyroid cancer.

**Methods:**

Univariate analysis was performed to identify potential indicators associated with intraoperative hypothermia. Multivariable logistic regression analysis was employed to select the independent predictors for model construction. The predictive performance and clinical utility of the model were assessed using receiver operating characteristic (ROC) curve, calibration plots and decision curve analysis (DCA). External validation was conducted to evaluate its generalizability.

**Results:**

Univariate analysis revealed that age, body mass index (BMI), anesthesia duration, duration of surgery, infusion volume, intraoperative irrigation volume, irrigation fluid temperature and intraoperative blood loss were significantly associated with the occurrence of intraoperative hypothermia (all P < 0.05). Multivariate logistic regression analysis identified infusion volume and irrigation fluid temperature were independent risk factors for intraoperative hypothermia in patients undergoing endoscopic radical thyroidectomy for thyroid cancer, whereas BMI was an independent protective factor (P < 0.05). ROC curve indicated excellent predictive accuracy of the model (AUC = 0.945). The calibration plot demonstrated a high degree of concordance between the actual incidence and the predicted probabilities. The results of DCA indicated that this predictive model has high clinical application value. When applied to the validation cohort, the model maintained strong predictive performance and stability, with an AUC of 0.831.

**Conclusion:**

The nomogram model developed in this study exhibits strong predictive performance and high clinical utility in assessing the risk of intraoperative hypothermia among patients undergoing endoscopic thyroid cancer radical surgery, serving as a valuable reference for operating room nurses in identifying high-risk individuals.

## 1 Introduction

Thyroid cancer is one of the most common endocrine malignancies. Papillary thyroid carcinoma and follicular thyroid carcinoma together constitute over 90% of all thyroid carcinoma cases (Prete et al., 2020). Most patients with these subtypes have an excellent prognosis, with more than 90% achieving disease-specific survival beyond 10 years (Jemal et al., 2009). The incidence of thyroid carcinoma has increased significantly over the past 30 years (Kim et al., 2020). Young women represent a high-risk population for thyroid cancer, accounting for approximately 7.5%–10% of all cases (Bleyer et al., 2006). Therefore, during surgical management, clinicians should aim not only for complete disease eradication but also for optimal postoperative cosmetic outcomes to address patients’ dual concerns regarding functional integrity and aesthetic appearance (Sephton, 2019). In recent years, with advances in technology, endoscopic radical resection for thyroid cancer has been increasingly adopted due to its advantages, including improved surgical visualization, minimal incisions, and aesthetically concealed incision sites (Li et al., 2023). However, because this procedure is technically complex and typically involves prolonged operative duration, 50%–70% of patients experience intraoperative hypothermia (Zou et al., 2020).

Intraoperative hypothermia refers to a decrease in the patient’s core body temperature below 36 °C for any reason during surgery, and it represents a common perioperative complication (Wang et al., 2022). Intraoperative hypothermia is correlated with multiple adverse consequences, such as cardiovascular complications following surgery, perioperative hemorrhage, disrupted drug metabolism, surgical wound infections, heightened risk of postoperative delirium, and a greater probability of deep vein thrombosis occurrence (Frank et al., 1997; Ju et al., 2023; Stöckler et al., 1989; Li et al., 2022). Furthermore, intraoperative hypothermia may also delay emergence from anesthesia (Pan et al., 2025), diminish thermal comfort and patient satisfaction, and lead to elevated healthcare spending (Soilly et al., 2023). Therefore, maintaining stable intraoperative body temperature is a critical intervention for reducing the risk of perioperative and anesthesia-related complications.

Numerous studies have demonstrated that intraoperative hypothermia is associated with a range of contributing factors. Anesthetic agents play a key role, particularly general anesthetics, which impair central thermoregulation, induce peripheral vasodilation, and consequently enhance heat loss (Cho et al., 2024). Environmental and procedural elements further contribute to hypothermia risk, including low ambient operating room temperatures, prolonged patient exposure to cold environments, and the administration of unwarmed irrigation or intravenous fluids (Campbell et al., 2015). Moreover, longer surgical duration and greater exposure of body cavities increase the surface area for heat dissipation, thereby elevating the likelihood of significant thermal loss (Zhao et al., 2023). Furthermore, the incidence of hypothermia varies across surgical types, being notably higher in major abdominal procedures, trauma surgeries, and extended laparoscopic operations (Shen and He, 2024). Therefore, systematic identification of these risk factors and the development of tailored perioperative temperature management strategies are essential for reducing the occurrence of intraoperative hypothermia and its associated complications.

In recent years, the rapid advancement of artificial intelligence has driven a growing number of studies on intraoperative hypothermia prediction models, offering robust support for early and accurate risk stratification of intraoperative hypothermia (Jiang et al., 2024; Huang et al., 2025). However, research on predictive models for intraoperative hypothermia in patients undergoing endoscopic radical thyroidectomy remains limited, and the development and validation of these models necessitate systematic investigation and rigorous clinical evaluation. Here, we identified key risk factors associated with hypothermia during endoscopic radical thyroidectomy and developed a predictive model based on these variables. The model demonstrates strong discriminatory capacity in identifying high-risk patients, thereby offering a reliable and evidence-based foundation for timely intraoperative hypothermia prevention and management.

## 2 Materials and methods

### 2.1 Study design

The study is a retrospective study and follows TRIPOD statement. A total of 280 patients who underwent endoscopic radical thyroidectomy at Jiangsu Provincial People’s Hospital between January 2022 and January 2023 were included in the training cohort. An independent external validation cohort comprising 120 patients who underwent the same procedure at Jiangsu Cancer Hospital during the same period was established, thereby enhancing the model’s generalizability and clinical applicability. Inclusion criteria were as follows: patients aged ≥18 years who had complete clinical characteristic data. The nasopharynx was used as the site for core body temperature monitoring (Bindu et al., 2017). Body temperature data were continuously collected at each time point from the induction of anesthesia until patient transfer out of the operating room using an anesthesia monitor. A patient was classified as having intraoperative hypothermia if body temperature dropped to <36 °C at any time during this period.

### 2.2 Sample

Based on a comprehensive literature review and expert consensus (Cho et al., 2022; Tavares Mendonça et al., 2021), this study incorporated 16 potential risk factors. It has been widely reported that a sample size of 10–15 times the number of independent variables is necessary to ensure reliable parameter estimation. Considering an expected sample loss rate of 10%–20%, the required sample size ranges from 176 to 288 cases. Therefore, the 253 cases in the training cohort used for modeling satisfy the sample size requirements.

### 2.3 Clinical data collection

All data were extracted from patients’ medical records, nursing records, and anesthesia record sheets using a self-designed data collection form. The organized data has been encoded according to the preset rules as shown in Table 1.

**TABLE 1:** List of variable assignments.

Variables	Assignment
Intraoperative temperature	0 indicates intraoperative temperature ≥36.0 °C, 1 indicates <36.0 °C
Gender	0 indicates female, 1 indicates male
Age (years)	0 indicates <30, 1 indicates 30–60, and 2 indicates >60
BMI (kg/m^2^)	0 indicates <18.5, 1 indicates 18.5–23.9, and 2 indicates >23.9
History of underlying diseases (diabetes or hypertensive)	0 indicates no, 1 indicates yes
HR (beats/min)	0 indicates <60, 1 indicates 60–100, and 2 indicates >100
Preoperative blood pressure (mmHg)	0 indicates low, values < 90/60, 1 indicates normal, values = 90–140/60–90, and 2 indicates high, values >140/90
Basal body temperature	0 indicates <36.5 °C, 1 indicates 36.5–37 °C, and 2 indicates >37.0 °C
Operating room temperature	0 indicates ≥23.0 °C, 1 indicates <23.0 °C
ASA	0 indicates grade 1, 1 indicates grade 2, and 2 indicates grade 3
Anesthesia duration (min)	0 indicates <120, 1 indicates 120–180, and 2 indicates >180
Duration of surgery (min)	0 indicates <120, 1 indicates 120–180, and 2 indicates >180
Intraoperative infusion volume (mL)	0 indicates <1,000, 1 indicates 1,000–2000, and 2 indicates >2000
Intraoperative irrigation volume (mL)	0 indicates ≥2000, 1 indicates <2000
Irrigation fluid temperature	0 indicates room temperature, 1 indicates constant temperature (37 °C)
Intraoperative blood loss (mL)	0 indicates ≥100, 1 indicates <100
Blood transfusion	0 indicates no, 1 indicates yes

Abbreviation: BMI, body mass index; HR, heart rate; ASA, american society of anesthesiologists.

### 2.4 Model construction and validation

This study first employed univariate analysis to conduct a preliminary screening of the association between independent variables and intraoperative hypothermia, in order to identify potential influencing factors with statistical significance. Subsequently, significant variables identified through the logistic regression analysis were incorporated as predictors into the predictive model. R language was used to build a postoperative hypothermia prediction model. Model performance was assessed using receiver operating characteristic (ROC) curves, calibration curves, and decision curve analysis (DCA), with external validation performed on the validation set. Modeling is achieved through the use of “readxl”, “pROC”, “rms”, “dcurves” and “dplyr” R packages.

### 2.5 Statistical analysis

All data were analyzed using SPSS (version 28.0) and R software (version 3.6.1). Categorical variables are reported as frequency and percentage (%), and group comparisons were conducted using the chi-square (χ^2^) test. The p value <0.05 was considered statistically significant.

## 3 Results

### 3.1 Study participant selection and flow diagram

**FIGURE 1:**
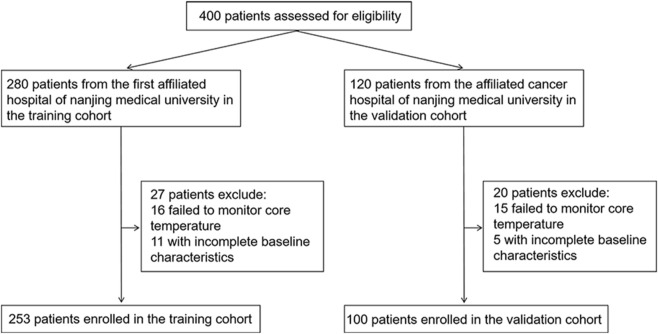
Flowchart of the study.

A total of 400 patients who met the inclusion criteria were enrolled in this study. Of these, 280 patients were recruited from Jiangsu Provincial People’s Hospital and constituted the training cohort, while 120 patients were recruited from Jiangsu Cancer Hospital and formed the external validation cohort. In the training cohort, 16 patients with incomplete temperature monitoring data and 11 with incomplete baseline data were excluded, resulting in a final inclusion of 253 patients. In the validation cohort, 15 patients with incomplete temperature monitoring data and 5 with incomplete baseline data were excluded, yielding a final sample of 100 patients. The detailed patient selection process is illustrated in Figure 1.

### 3.2 Baseline characteristics of the study population

A total of 253 patients were included in the training cohort, of whom 109 experienced intraoperative hypothermia and 144 maintained normal body temperature during surgery. The overall incidence of intraoperative hypothermia was 43.08%. More sample characteristics are shown in Table 2. Univariate analysis revealed that age, BMI, anesthesia duration, duration of surgery, infusion volume, intraoperative irrigation volume, irrigation fluid temperature and intraoperative blood loss were significantly associated with the occurrence of intraoperative hypothermia (Table 2).

**TABLE 2:** Differences in demographic and clinical characteristics between the hypothermia and Non-hypothermia groups in the training cohort.

Demographic characteristics [n (%)]	Non-hypothermia group (n = 144)	Hypothermia group (n = 109)	Statistical value	*P* value
Gender			0.310	0.577
Female	83 (57.64)	59 (54.13)		
Male	61 (42.36)	50 (45.87)		
Age (years)			12.851	**0.002**
<30	18 (12.50)	6 (5.50)		
30–60	52 (45.61)	23 (21.10)		
>60	74 (51.39)	80 (73.39)		
BMI (kg/m^2^)			23.267	**<0.001**
<18.5	25 (17.36)	49 (44.95)		
18.5–23.9	73 (50.69)	40 (36.70)		
>23.9	46 (31.94)	20 (18.35)		
History of underlying diseases			1.435	0.231
No	54 (37.50)	33 (30.28)		
Yes	90 (62.50)	76 (69.72)		
Heart rate (beats/min)			1.393	0.498
<60	7 (4.86)	3 (2.75)		
60–100	132 (91.67)	104 (95.41)		
>100	5 (3.47)	2 (1.83)		
Preoperative blood pressure (mmHg)			0.754	0.686
Low	12 (8.33)	6 (5.50)		
Normal	122 (84.72)	95 (87.16)		
High	10 (6.94)	8 (7.34)		
Basal body temperature			2.671	0.263
<36.5 °C	84 (58.33)	57 (52.29)		
36.5–37 °C	49 (34.03)	47 (43.12)		
>37.0 °C	11 (7.64)	5 (4.59)		
Operating room temperature			0.001	0.976
≥23.0 °C	127 (88.19)	96 (88.07)		
<23.0 °C	17 (11.81)	13 (11.93)		
ASA			0.957	0.620
Grade 1	5 (3.47)	6 (5.50)		
Grade 2	98 (68.06)	69 (63.30)		
Grade 3	41 (28.47)	34 (31.19)		
Anesthesia duration (min)			21.919	**<0.001**
<120	37 (25.69)	22 (20.18)		
120–180	82 (56.94)	39 (35.78)		
>180	25 (17.36)	48 (44.04)		
Duration of surgery (min)			18.757	**<0.001**
<120	38 (26.39)	29 (26.61)		
120–180	87 (60.42)	42 (38.53)		
>180	19 (13.19)	38 (34.86)		
Infusion volume (mL)			59.275	**<0.001**
<1,000	42 (29.17)	6 (5.50)		
1,000–2000	79 (54.86)	37 (33.94)		
>2000	23 (15.97)	66 (60.55)		
Intraoperative irrigation volume (mL)			5.116	**0.024**
≥2000	25 (17.36)	32 (29.36)		
<2000	119 (82.64)	77 (70.64)		
Irrigation fluid temperature			21.001	**<0.001**
Room temperature	35 (24.31)	57 (52.29)		
Constant temperature (37 °C)	109 (75.69)	52 (47.71)		
Intraoperative blood loss (mL)			6.461	**0.011**
≥100	76 (52.78)	40 (36.70)		
<100	68 (47.22)	69 (63.30)		
Blood transfusion			0.161	0.688
No	140 (97.22)	105 (96.33)		
Yes	4 (2.78)	4 (3.67)		

Abbreviation: BMI, body mass index; ASA, american society of anesthesiologists. The bold value indicates that the result is statistically significant.

### 3.3 Feature selection and model construction

To identify the independent predictors, the eight variables significantly associated with intraoperative hypothermia were entered into a multivariate logistic regression model. As shown in Table 3, logistic regression analysis showed that infusion volume and irrigation fluid temperature were independent risk factors for intraoperative hypothermia in patients undergoing endoscopic radical thyroidectomy for thyroid cancer, whereas BMI was an independent protective factor (P < 0.05).

**TABLE 3:** Predictors of hypothermia in patients undergoing endoscopic radical thyroidectomy for thyroid cancer.

Variables	*β*	Standard error	Wald value	*OR*	95% *CI*	*P* vaule
Age	0.036	0.019	3.447	1.036	0.998–1.076	0.063
BMI	−1.053	0.222	22.399	0.349	0.226–0.540	**<0.001**
Anesthesia duration	0.011	0.035	0.097	1.011	0.944–1.083	0.755
Duration of surgery	−0.058	0.034	2.795	0.944	0.882–1.010	0.095
Infusion volume	7.917	2.026	15.276	8.115	3.474–21.182	**<0.001**
Intraoperative irrigation volume	0.805	1.033	0.608	2.236	0.296–16.925	0.436
Irrigation fluid temperature	3.417	0.823	17.224	30.486	6.070–153.100	**<0.001**
Intraoperative blood loss	0.872	1.074	0.633	1.089	0.988–1.190	0.412

β is the regression coefficient. Abbreviation: BMI, body mass index; CI, confidence interval; OR, odds ratio. The bold value indicates that the result is statistically significant.

**FIGURE 2:**
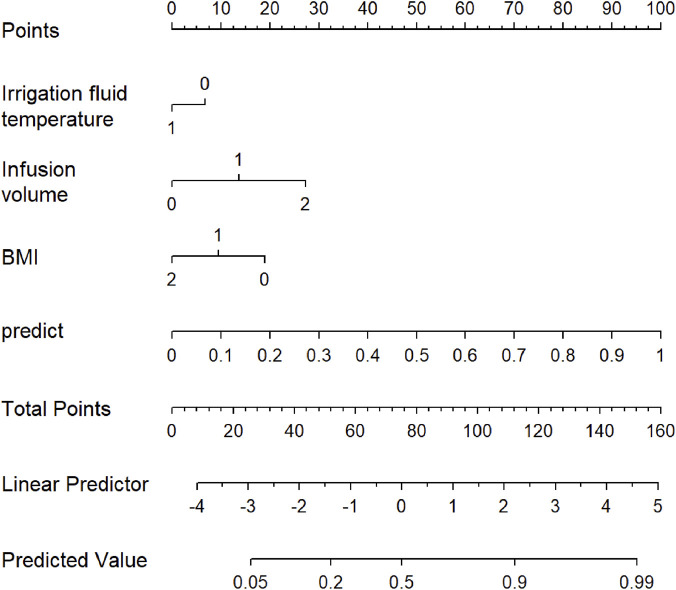
Developed intraoperative hypothermia nomogram. The intraoperative hypothermia nomogram was developed in the training cohort, with irrigation fluid temperature, infusion volume and BMI. BMI: body mass index.

Subsequently, independent risk factors associated with intraoperative hypothermia in patients undergoing endoscopic radical thyroidectomy were systematically integrated into R software to develop a nomogram-based predictive model, facilitating individualized risk assessment and clinical decision-making. This nomogram model is shown in Figure 2.

### 3.4 Evaluation of the predictive value of the model

**FIGURE 3:**
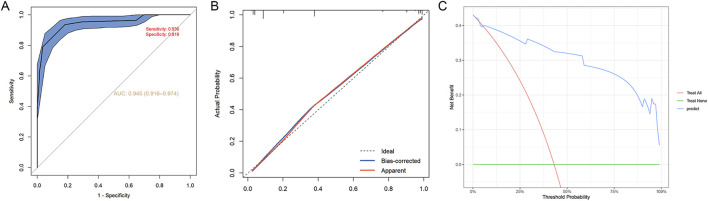
Predictive nomogram model evaluation. **(A)** ROC curve, **(B)** calibration plots and **(C)** decision curve analysis of the intraoperative hypothermia nomogram prediction in the training cohort. ROC, Receiver operating characteristic.

Internal validation of the nomogram model was performed using the Bootstrap method with 1,000 resampling iterations to assess its stability (Xia et al., 2024). The predictive accuracy of the model for intraoperative hypothermia in patients with thyroid cancer undergoing endoscopic surgery was assessed using ROC curve analysis. The area under the curve (AUC) was 0.945 [95% CI (0.916–0.974)], with a sensitivity of 0.936 and a specificity of 0.819, indicating that the risk prediction model exhibited good discriminatory performance and could effectively stratify the risk of intraoperative hypothermia in patients undergoing endoscopic thyroidectomy (Figure 3A). The calibration curve demonstrates good agreement between the observed and predicted probabilities, further confirming the accuracy of the model (Figure 3B).

In addition, to assess the clinical value of the nomogram model, the DCA method was used. DCA evaluates the clinical utility of predictive models by quantifying the net benefit across a range of threshold probabilities in a patient population (Mei et al., 2022). As shown in Figure 3C, this model demonstrates a higher net benefit across a range of threshold probabilities, indicating significant clinical advantages.

### 3.5 External validation

A total of 100 patients were included in the validation cohort, of whom 38 experienced intraoperative hypothermia and 62 maintained normal body temperature during surgery. The overall incidence of intraoperative hypothermia was 38%. In contrast to the training cohort, the validation cohort results demonstrated that gender and basal body temperature were significantly associated with the occurrence of intraoperative hypothermia, whereas intraoperative blood loss showed a weaker association (Table 4).

**TABLE 4:** Differences in demographic and clinical characteristics between the hypothermia and Non-hypothermia groups in validation cohort.

Demographic characteristics [n (%)]	Non-hypothermia group (n = 62)	Hypothermia group (n = 38)	Statistical value	*P* value
Gender			6.629	**0.010**
Female	35 (56.45)	31 (81.58)		
Male	27 (43.55)	7 (18.42)		
Age (years)			15.382	**<0.001**
<30	11 (17.74)	0 (0.00)		
30–60	16 (25.81)	23 (60.53)		
>60	35 (56.45)	15 (39.47)		
BMI (kg/m^2^)			7.678	**0.022**
<18.5	17 (27.42)	7 (18.42)		
18.5–23.9	32 (51.61)	13 (34.21)		
>23.9	13 (20.97)	18 (47.37)		
History of underlying diseases			1.181	0.277
No	21 (33.87)	17 (44.74)		
Yes	41 (66.13)	21 (55.26)		
Heart rate (beats/min)			3.879	0.144
<60	6 (9.68)	1 (2.63)		
60–100	53 (85.48)	37 (97.37)		
>100	3 (4.84)	0 (0.00)		
Preoperative blood pressure (mmHg)			1.040	0.594
Low	9 (14.52)	3 (7.89)		
Normal	46 (74.19)	31 (81.58)		
High	7 (11.29)	4 (10.53)		
Basal body temperature			19.106	**<0.001**
<36.5 °C	20 (32.26)	29 (76.32)		
36.5–37 °C	35 (56.45)	6 (15.79)		
>37.0 °C	7 (11.29)	3 (7.89)		
Operating room temperature			0.614	0.433
≥23.0 °C	52 (83.87)	34 (89.47)		
<23.0 °C	10 (16.13)	4 (10.53)		
ASA			1.213	0.545
Grade 1	18 (29.03)	8 (21.05)		
Grade 2	41 (66.13)	29 (76.32)		
Grade 3	3 (4.84)	1 (2.63)		
Anesthesia duration (min)			21.294	**<0.001**
<120	23 (37.10)	5 (13.16)		
120–180	30 (48.39)	11 (28.95)		
>180	9 (14.52)	22 (57.89)		
Duration of surgery (min)			9.012	**0.011**
<120	23 (37.10)	7 (18.42)		
120–180	31 (50.00)	17 (44.74)		
>180	8 (12.90)	14 (36.84)		
Infusion volume (mL)			19.458	**<0.001**
<1,000	24 (38.71)	2 (5.26)		
1,000–2000	28 (45.16)	17 (44.74)		
>2000	10 (16.13)	19 (50.00)		
Intraoperative irrigation volume (mL)			9.564	**0.002**
≥2000	9 (14.52)	16 (42.11)		
<2000	53 (85.48)	22 (57.89)		
Irrigation fluid temperature			16.352	**<0.001**
Room temperature	6 (9.68)	17 (44.74)		
Constant temperature (37 °C)	56 (90.32)	21 (55.26)		
Intraoperative blood loss (mL)			0.075	0.784
≥100	36 (58.06)	21 (55.26)		
<100	26 (41.94)	17 (44.74)		
Blood transfusion			0.255	0.614
No	60 (96.77)	36 (94.74)		
Yes	2 (3.23)	2 (5.26)		

Abbreviation: BMI, body mass index; ASA, american society of anesthesiologists. The bold value indicates that the result is statistically significant.

Subsequently, a statistical analysis was performed on the 16 clinical characteristics in both the training and validation sets. As shown in Table 5, apart from american society of anesthesiologists (ASA) classification and irrigation fluid temperature, all other variables showed no statistically significant differences. It is possible that the difference of irrigation fluid temperature contributes to the slightly lower incidence of intraoperative hypothermia observed in the validation cohort compared to the training cohort.

**TABLE 5:** Comparison of clinical characteristics between the training cohort and the validation cohort.

Demographic characteristics [n (%)]	Training cohort (n = 253)	Validation cohort (n = 100)	Statistical value	*P* value
Status			0.762	0.383
Hypothermia	109 (43.08)	38 (38.00)		
Non-hypothermia	144 (56.92)	62 (62.00)		
Gender			2.887	0.089
Female	142 (56.13)	66 (66.00)		
Male	111 (43.87)	34 (34.00)		
Age (years)			3.573	0.168
<30	24 (9.49)	11 (11.00)		
30–60	75 (29.64)	39 (39.00)		
>60	154 (60.87)	50 (50.00)		
BMI (kg/m^2^)			1.343	0.511
<18.5	74 (29.25)	24 (24.00)		
18.5–23.9	113 (44.66)	45 (45.00)		
>23.9	66 (26.09)	31 (31.00)		
History of underlying diseases			0.409	0.522
No	87 (34.39)	38 (38.00)		
Yes	166 (65.61)	62 (62.00)		
Heart rate (beats/min)			1.479	0.477
<60	10 (3.95)	7 (7.00)		
60–100	236 (93.28)	90 (90.00)		
>100	7 (2.77)	3 (3.00)		
Preoperative blood pressure (mmHg)			3.992	0.136
Low	18 (7.11)	12 (12.00)		
Normal	217 (85.77)	77 (77.00)		
High	18 (7.11)	11 (11.00)		
Basal body temperature			2.091	0.352
<36.5 °C	141 (55.73)	49 (49.00)		
36.5–37 °C	96 (37.94)	41 (41.00)		
>37.0 °C	16 (6.32)	10 (10.00)		
Operating room temperature			0.301	0.583
≥23.0 °C	30 (11.86)	14 (14.00)		
<23.0 °C	223 (88.14)	86 (86.00)		
ASA			53.288	**<0.001**
Grade 1	11 (4.35)	26 (26.00)		
Grade 2	167 (66.01)	70 (70.00)		
Grade 3	75 (29.64)	4 (4.00)		
Anesthesia duration (min)			1.477	0.478
<120	59 (23.32)	28 (28.00)		
120–180	121 (47.83)	41 (41.00)		
>180	73 (28.85)	31 (31.00)		
Duration of surgery (min)			0.459	0.795
<120	67 (26.48)	30 (30.00)		
120–180	129 (50.99)	48 (48.00)		
>180	57 (22.53)	22 (22.00)		
Infusion volume (mL)			2.518	0.284
<1,000	48 (18.97)	26 (26.00)		
1,000–2000	89 (35.18)	45 (45.00)		
>2000	116 (45.85)	29 (29.00)		
Intraoperative irrigation volume (mL)			0.245	0.620
≥2000	196 (77.47)	75 (75.00)		
<2000	57 (22.53)	25 (25.00)		
Irrigation fluid temperature			5.827	**0.016**
Room temperature	92 (36.36)	23 (23.00)		
Constant temperature (37 °C)	161 (63.64)	77 (77.00)		
Intraoperative blood loss (mL)			3.566	0.059
≥100	137 (54.15)	57 (57.00)		
<100	116 (45.85)	43 (43.00)		
Blood transfusion			0.153	0.695
No	245 (96.84)	96 (96.00)		
Yes	8 (3.16)	4 (4.00)		

Abbreviation: BMI, body mass index; ASA, american society of anesthesiologists. The bold value indicates that the result is statistically significant.

**FIGURE 4:**
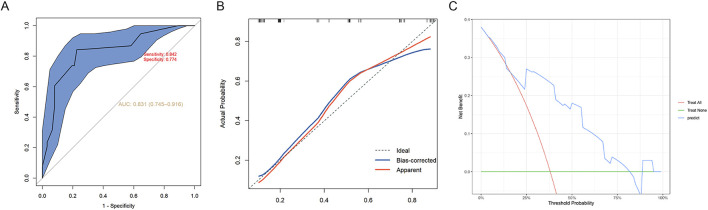
Model validation by external data. **(A)** ROC curve, **(B)** calibration plots and **(C)** decision curve analysis of the intraoperative hypothermia nomogram prediction in the validation cohort. ROC, Receiver operating characteristic.

To evaluate the stability and applicability of the constructed predictive model, we conducted an external validation of it. The ROC curve yielded an AUC of 0.831, with a sensitivity of 0.842 and a specificity of 0.774 (Figure 4A). The calibration curves showed good agreement between predicted and observed probabilities, with both internally and externally corrected curves closely aligning with the ideal diagonal line (Figure 4B). The results of DCA also indicated that this model has high clinical application value (Figure 4C).

## 4 Discussion

The incidence of thyroid cancer is increasing significantly, particularly among female populations (Wiltshire et al., 2016; Chen et al., 2018). Surgery remains one of the primary treatment modalities for thyroid cancer (Kaliszewski et al., 2020). Endoscopic radical thyroidectomy, as a representative minimally invasive technique, has been widely adopted in clinical practice due to its favorable safety profile, rapid postoperative recovery, and reduced pain intensity (Bian et al., 2018). However, this procedure requires general anesthesia, which may impair the patient’s thermoregulatory function (Daniel, 2008). Furthermore, intraoperative administration of large volumes of unwarmed intravenous fluids and prolonged exposure of the patient to the cool operating room environment collectively contribute to an increased risk of intraoperative hypothermia (Leslie and Sessler, 2003). Therefore, identifying high-risk patients for intraoperative hypothermia among individuals with thyroid cancer undergoing endoscopic radical surgery, and proactively implementing evidence-based thermal protection nursing interventions, can enhance surgical outcomes and facilitate postoperative recovery.

Currently, nomograms have been widely applied in the fields of oncology and internal medicine due to their user-friendly digital interfaces, higher predictive accuracy, and intuitive expression of prognosis information, becoming an important tool for assisting clinical decision-making. Dong et al. (2022) developed a nomogram to predict the risk of postoperative delirium in patients undergoing laparoscopic surgery for gynecologic cancers. The model exhibited robust predictive accuracy, with an area under the receiver operating characteristic curve of 0.833. Additionally, previous studies have demonstrated the application of nomogram models in predicting the risk of intraoperative hypothermia across various surgical procedures, including laparoscopic radical resection for colorectal cancer (Yan et al., 2023), video-assisted thoracoscopic lobectomy (Xia et al., 2025), and lung transplantation (Huang et al., 2024), among others. However, research on predictive models for intraoperative hypothermia in patients undergoing endoscopic radical thyroidectomy remains limited. This study represents the first application of nomograms in endoscopic thyroid surgery for predicting the risk of intraoperative hypothermia, offering a novel quantitative approach to perioperative temperature management.

In the study, age, BMI, anesthesia duration, duration of surgery, infusion volume, intraoperative irrigation volume, irrigation fluid temperature and intraoperative blood loss were found to be closely related to the occurrence of intraoperative hypothermia in training cohort. The results exhibit a slight discrepancy compared to the validation cohort, which may be attributed to differences in data sources. Further research indicates that fluid infusion volume and irrigation fluid temperature are independent risk factors for intraoperative hypothermia, whereas BMI serves as an independent protective factor.

Due to preoperative fasting and substantial intraoperative fluid loss, large volumes of room-temperature or refrigerated intravenous fluids and blood products are commonly administered to restore circulating blood volume, maintain internal homeostasis, and ensure hemodynamic stability. However, the infusion of large quantities of such fluids not only increases metabolic heat demand but also results in direct thermal loss due to their subphysiological temperatures, thereby contributing to the development of intraoperative hypothermia (Okada et al., 2020). Similar to previous studies, the passage of irrigation fluid through the patient’s body contributes to increased heat loss, thereby predisposing patients to shivering and intraoperative hypothermia at room temperature (Liu et al., 2025). Moreover, the present study demonstrates that a higher BMI is linked to a protective effect against hypothermia in patients, consistent with evidence from prior research (Yi et al., 2017). According to the gradient theory, heat produced by metabolic activity is transferred from the core region to peripheral tissues and subsequently dissipated into the external environment (Emmert et al., 2018). Therefore, a higher BMI may confer protective benefits, as adipose tissue serves as an effective thermal insulator, attenuating heat loss during the conduction process (Groene et al., 2019).

Based on the above three important factors, the study developed a highly accurate prediction model for intraoperative hypothermia in patients undergoing endoscopic thyroidectomy for cancer. External validation demonstrated strong discriminatory ability and excellent calibration, indicating that the nomogram has robust applicability and high precision in similar surgical settings. For patients at high risk of intraoperative hypothermia, the implementation of comprehensive, individualized warming interventions is recommended to effectively mitigate the likelihood of temperature decline during surgery.

It should be pointed out that this study has certain limitations. Firstly, this study is retrospective in design, and the analysis of selected risk factors did not encompass all variables potentially influencing intraoperative hypothermia. Certain plausible factors (such as psychological stress or preoperative anxiety) were not captured during data collection. Future efforts will focus on expanding data acquisition to include these and other overlooked variables, enabling iterative refinement and enhanced generalizability of the predictive model. Secondly, the sample size was limited, lacking large-scale data to further verify the stability and generalization ability of the prediction model. Subsequent multi-center large data validation is needed. Thirdly, the study is limited by a narrow methodological approach. Future work will incorporate advanced machine learning techniques to enable a more comprehensive evaluation of the model’s performance and predictive robustness. Finally, unlike previous research results, this study did not list age and comorbidities as risk factors, and this difference needs to be confirmed in future studies (Ozaki et al., 1997). Therefore, it is still necessary to conduct well-designed, multi-center, large-sample studies to further improve the accuracy and clinical applicability of the risk prediction model.

## 5 Conclusion

In conclusion, this study developed a nomogram model with excellent predictive performance, which can assist nursing staff in assessing the risk of intraoperative hypothermia among patients undergoing endoscopic thyroid surgery. This capability enables timely implementation of proactive warming interventions to maintain stable intraoperative body temperature.

## Data Availability

The raw data supporting the conclusions of this article will be made available by the authors, without undue reservation.
